# Chaotic Teaching-Learning-Based Optimization with Lévy Flight for Global Numerical Optimization

**DOI:** 10.1155/2016/8341275

**Published:** 2016-01-31

**Authors:** Xiangzhu He, Jida Huang, Yunqing Rao, Liang Gao

**Affiliations:** ^1^State Key Laboratory of Digital Manufacturing Equipment and Technology, Huazhong University of Science and Technology, Wuhan 430074, China; ^2^College of Electronics and Information Engineering, South-Central University for Nationalities, Wuhan 430074, China

## Abstract

Recently, teaching-learning-based optimization (TLBO), as one of the emerging nature-inspired heuristic algorithms, has attracted increasing attention. In order to enhance its convergence rate and prevent it from getting stuck in local optima, a novel metaheuristic has been developed in this paper, where particular characteristics of the chaos mechanism and Lévy flight are introduced to the basic framework of TLBO. The new algorithm is tested on several large-scale nonlinear benchmark functions with different characteristics and compared with other methods. Experimental results show that the proposed algorithm outperforms other algorithms and achieves a satisfactory improvement over TLBO.

## 1. Introduction

Optimization problems are always associated with many kinds of difficult characteristics involving multimodality, dimensionality, and differentiability [1]. Traditional methods like linear programming and dynamic programming generally fail to optimize such problems especially when these problems have nonlinear objective functions, as most of these traditional techniques require gradient information and easily converge to local optima. Moreover, those classical search approaches depend heavily on variables and functions, which prevents them from yielding a generalized and flexible solution scheme, especially for large-scale and nonlinear optimization [2]. Under this circumstance, swarm intelligence, which deals with the collective behavior of swarms through complex interaction of individuals without supervision, has become a hot research area [3]. The inherent strengths of the swarm optimization techniques, including fault tolerance, adaptation, speed, autonomy, and parallelism [4], allow them to be applied more effectively and widely compared with the previous algorithms [5].

Several well-known swarm algorithms have been proposed in the latest years. For example, ant colony optimization (ACO) is based on the metaphor of ants seeking food [6]. Particle swarm optimization (PSO) works on the foraging behavior of a biological social system like a flock of birds [7]. Artificial bee colony (ABC) simulates the intelligent foraging behavior of a honeybee [8]. These algorithms have been applied to many engineering optimization problems and proved effective in solving some specific kind of problems.

Teaching-learning-based optimization (TLBO) algorithm is a teaching-learning inspired algorithm proposed by Rao et al., which is based on the effect of influence of a teacher on the output of learners in a class [9, 10]. The TLBO is free from parameters and has been compared with other well-known optimization algorithms such as PSO [11]. The results show better performance of TLBO over other methods. Applications of this algorithm have also been widely tested in different optimization fields; for example, Toĝan [12] employed the TLBO algorithm in the discrete optimization of planar steel frames and found that TLBO is a more powerful optimization method than other algorithms like Genetic Algorithm (GA), ACO, and Harmony Search (HS). Amiri [13] similarly applied TLBO to solve clustering problems and verified the robustness and flexibility of this method. However, simulation results from Huang et al. showed that TLBO could not obtain satisfactory results for several difficult benchmark problems which have complex landscapes and was prone to becoming trapped in locally optimal solutions [14]. To overcome this technical barrier, Rao and Patel modified many aspects of the basic TLBO such as incorporating an elitism strategy in it, using adaptive teaching factor and multiteacher approaches to improve its performance [15]. Based on some insight into the structure of TLBO, we also found that it lacks diversification because it only calculates the mean value of the population and searches between two randomly chosen individual solutions in the search iterations.

Chaos is a universal phenomenon of nonlinear dynamic systems, which has been extensively studied since Lorent [16] discovered the authoritative chaotic attractor in 1963. Chaos is a bounded unstable dynamic behavior that exhibits sensitive dependence on initial conditions and includes infinite unstable periodic motions. Although it appears to be stochastic, it occurs in a deterministic nonlinear system under deterministic conditions [17]. Due to its properties, chaos has been applied to many kinds of areas of optimization computation [18, 19]. Zuo and Fan [20] proposed the chaos search immune algorithm and applied it to neurofuzzy controller design. Alatas et al. used the chaotic search to improve the performance of PSO algorithms [21] and proposed chaotic bee colony algorithms [22]. Chuang et al. [23] proposed chaotic catfish PSO.

Lévy flight is another technique for speeding up the convergence rate of the algorithm and escaping from local optima [24]. As a typical flight behavior of many animals and insects, Lévy flight was originally researched by Lévy and Borel in 1954 [25] and has been subsequently used for nonlocal searches in many optimization problems due to its promising capability [26, 27]. Since the step length of the random walk produced by Lévy flight is drawn from a power-law distribution with a heavy tail, namely, Lévy distribution, part of the new population is generated near the current best solution, and therefore this technique can speed up the local search. Further, most of the new solutions are produced far from the current best solution, which prevents the algorithm from becoming trapped in local optima.

An efficient optimization algorithm means it has both strong exploration ability and a fast exploitation rate; moreover, the method can be adapted to tackle a broad range of problems [28]. In order to reinforce the performance of the TLBO and broaden the diversification of the algorithm, a chaotic system and Lévy flight mechanism are introduced into the TLBO. The basic idea of the proposed algorithm is as follows. First, the population in the TLBO is divided into two parts according to the fitness of the solutions in the population. Then a Lévy flight is performed on the worse part, while using the original teaching-learning search mechanism for the better part. Secondly, the chaotic search is implemented on a randomly chosen part of the population for the sake of diversity. The numerical experiments demonstrate the effectiveness of the proposed algorithm.

This paper is organized as follows. In Section 2, the basic TLBO is introduced. Then the proposed chaotic TLBO with Lévy flight is presented in Section 3. In Section 4, some experiments are performed and the numerical results are shown. Finally, the conclusion of the paper is presented in Section 5.

## 2. Teaching-Learning-Based Optimization

TLBO is a recently published population-based method, which mimics the classic teaching-learning phenomenon within a classroom environment. In this novel optimization algorithm a group of learners is considered as population and different design variables are considered as different subjects offered to the learners and learners' result is analogous to the fitness value of the optimization problem. In the entire population the best solution is considered as the teacher. The main procedure of TLBO consists of two phases: teacher phase and learner phase. These two phases will be explained in the following parts.

### 2.1. Teacher Phase

This is the first stage of the algorithm where learners learn from the teacher. During this phase a teacher tries to increase the mean of the whole class to his or her level (the new mean). The difference between the existing mean and the new mean is given as

(1) $$\begin{array}{c} \text{Difference\_Mea}{\text{n}}_{i}=r_{i}\left(\;M_{new}-T_{F}M_{i}\;\right), \end{array}$$

where *M*_*i*_ is the mean of each design variable and *M*_new_ is the new mean for the *i*th iteration; within the equation, two randomly generated parameters are applied: *r*_*i*_ ranges between 0 and 1 and *T*_*F*_ is a teaching factor which can be either 1 or 2, thus influencing the value of the mean to be changed. In the algorithm, *T*_*F*_ plays a role of adjusting factor, which controls the moving direction and scale when updating solutions. The value of *T*_*F*_ is decided randomly with equal probability as

(2) $$\begin{array}{c} T_{F}=round\left[\;1+rand\left(\;0,1\;\right)\left\{\;2-1\;\right\}\;\right]. \end{array}$$

Based on this Difference_Mean, the existing solution is updated according to the following expression:

(3) $$\begin{array}{c} X_{new,i}=X_{old,i}+\text{Difference\_Mea}{\text{n}}_{i}. \end{array}$$

### 2.2. Learner Phase

It is the second part of the algorithm where learners increase their knowledge by interaction between themselves. A learner interacts randomly with another learner for enhancing his or her knowledge. A learner learns new things if the other one has more knowledge than him or her. Mathematically the learning phenomenon of this phase is expressed below.

At any iteration *i*, considering two different learners (solutions) *X*_*i*_ and *X*_*j*_, where *i* ≠ *j*,

(4) Xnew,i=Xold,i+riXj−Xiif  fXj<fXi,

(5) Xnew,i=Xold,i+riXi−Xjif  fXi<fXj.

*X*_new_ is accepted into the population if it gives a better function value.

The steps for implementing TLBO are as follows.

**Step 1 (define the optimization problem and initialize algorithm parameters).**

Initialize the population size (*P*_*n*_), number of design variables (*D*_*n*_), and number of generations (*G*_*n*_). Define the optimization problem as follows: minimize *f*(*X*), where *f*(*X*) is the objective function and *X* is a vector for design variables. Construct initial solutions according to *P*_*n*_ and *D*_*n*_.

**Step 2 (calculate *M*_*i*_ and *M*_new_).**

Calculate the mean of the population columnwise, which will give the mean of each design variable as *M*_*i*_. Identify the best solution (teacher) according to *X*_teacher_ = *X*_*f*(*X*)=min_; the teacher will try to move *M*_*i*_ to *X*_teacher_, so let *M*_new_ = *X*_teacher_.

**Step 3 .**

Calculate the Difference_Mean according to (1) by utilizing the teaching factor *T*_*F*_.

**Step 4 .**

Modify the solutions in the teacher phase based on (3) and accept the new solution if it is better than the existing one.

**Step 5 .**

Update the solution in the learner phase according to (4) and (5) and accept the better one into the population.

**Step 6 .**

Repeat Steps 2 to 5 until the termination criterion is met.

## 3. Chaotic Teaching-Learning-Based Optimization with Lévy Flight

An effective optimization algorithm must have a strong global searching ability along with a fast convergence rate. TLBO is free from specific algorithm parameters and outperforms PSO, HS, and so on due to its simplicity and efficiency. However, several hard benchmarks with complicated landscapes pose challenges to TLBO in finding a satisfactory result and escaping from local optima.

In order to enhance the performance of TLBO as well as take advantage of the properties of the chaotic system and Lévy flight, we integrate the chaotic search mechanism and Lévy flight into TLBO to improve its search efficiency. Hence, a chaotic TLBO with Lévy flight (CTLBO) is proposed in this paper. In the algorithm, the population is divided into two parts: the part with better fitness is evolved by the teaching-learning process in TLBO, while another part is performed with a Lévy flight. Then the chaotic perturbation is implemented on a randomly selected part of the population in terms of the diversification of the population. The main steps of CTLBO are elaborated in the next sections.

### 3.1. Lévy Flight

Lévy flights, also called Lévy motion, represent a kind of non-Gaussian stochastic process whose step sizes are distributed based on a Lévy stable distribution [25].

When generating new solutions *x* ^*t*+1^ for solution *i*, a Lévy flight is performed:

(6) $$\begin{array}{c} x_{i}^{t+1}=x_{i}^{t}+\alpha ⊕\text{Lévy}\left(\;\lambda \;\right), \end{array}$$

where *α* > 0 is the step size which is relevant to the scales of the problem. In most conditions, we let *α* = 1. The product ⊕ means entrywise multiplications [24]. Lévy flights essentially provide a random walk while their random steps are drawn from a Lévy distribution for large steps:

(7) $$\begin{array}{c} \text{Lévy}\left(\;\lambda \;\right)\sim u=t^{-\lambda },\;\left(1<\lambda \leq 3\right) \end{array}$$

which has an infinite variance with an infinite mean. Here the consecutive steps of a solution essentially form a random walk process which obeys a power-law step-length distribution with a heavy tail.

There are a few ways to implement Lévy flights; the method we chose in this paper is one of the most efficient and simple ways based on Mantegna algorithm; all the equations are detailed in [29].

### 3.2. Chaotic Search

Chaos is a deterministic, quasi-random process that is sensitive to the initial condition [30]. The nature of chaos is apparently random and unpredictable. Mathematically, chaos is randomness of a simple deterministic dynamical system and chaotic system can be considered as sources of randomness.

A chaotic map is a discrete-time dynamical system running in a chaotic condition [22]:

(8) $$\begin{array}{c} x_{k+1}=f\left(\;x_{k}\;\right),\;0<x_{k}<1,\;\;k=0,1,2,\ldots , \end{array}$$

where {*x*_*k*_:  *k* = 0,1, 2,…} is the chaotic sequence, which can be utilized as spread-spectrum sequence as random number sequence.

Chaotic sequences have been proved to be simple and fast to produce and reserve; it is unnecessary to store long sequences [31]. Only a few functions (chaotic maps) and parameters (initial conditions) are required even for very long sequences [22].

In this paper, *r* chaotic variables are generated by the following logistic mapping:

(9) $$\begin{array}{c} z_{i}^{j+1}=\mu_{i}z_{i}^{j}\left(\;1-z_{i}^{j}\;\right),\;i=1,2,\ldots ,r,\;\;j=1,2,\ldots , \end{array}$$

where *i* is the serial number of chaotic variables and *μ*_*i*_ = 4. Given the *r* chaotic variables and different initial values *z*_*i*_^0^ (*i* = 1,2,…, *r*), the values of the *r* chaotic variables *z*_*i*_^1^ (*i* = 1,2,…, *r*) are then produced by the logistic equation. Let *j* = 1,2,…, *N* − 1, and hence, other *N* − 1 solutions are produced by the same method.

### 3.3. Proposed Methods

By introducing the Lévy flight and the chaotic search into the TLBO, a new algorithm is proposed in this paper. The pseudocode of the proposed CTLBO is shown in Pseudocode 1.

## 4. Experimental Analysis and Numerical Results

In order to verify the performance of the proposed CTLBO and to analyze its properties, two sets of optimization problems are selected for the test experiments. In each set of problems, several well-known functions are used as benchmark problems to study the search behavior of the proposed CTLBO and to compare its performance with those of other algorithms.

### 4.1. Experiment 1

Firstly, to demonstrate the performance of the proposed algorithm, eight benchmark optimization problems [32] are selected as test functions. These eight benchmark functions were tested earlier with TLBO and improved TLBO by Rao and Patel [15]. The details of the benchmark functions are given in Table 1.

In [15], Rao and Patel tested all functions with 30000 maximum function evaluations. To maintain the consistency in the comparison, the CTLBO algorithm is also tested with the same maximum function evaluations. Each benchmark function undergoes 30 independent tests with CTLBO. The comparative results are in the form of the mean value and standard deviation of the objective function obtained after 30 independent runs, which are shown in Table 2.

It can be seen from Table 2 that the CTLBO achieved the global optimum value for the Sphere, Griewank, Weierstrass, Rastrigin, and NCRastrigin functions, and the CTLBO and I-TLBO algorithms perform equally well for these functions. For the Rosenbrock function, CTLBO performs better than the rest of the algorithms. For the Ackley function, the modified ABC algorithm performs better than the rest of the considered algorithms.

It can also be seen from Table 2 that the result of CTLBO for the Schwefel function is not as good as those of other functions. This is mainly because, in the teacher phase of TLBO, a mean value is used to update the solutions, and this mechanism may lead the solutions to the centre of a search region, but when the global minimum is not located in the centre of the feasible solution region, TLBO usually fails to find the global solution of the tested function. Actually this is one of the limitations of TLBO, and in CTLBO, part of the solution inherits this mechanism. Hence this is a potential weakness of the proposed algorithm.

**Figure 1:**
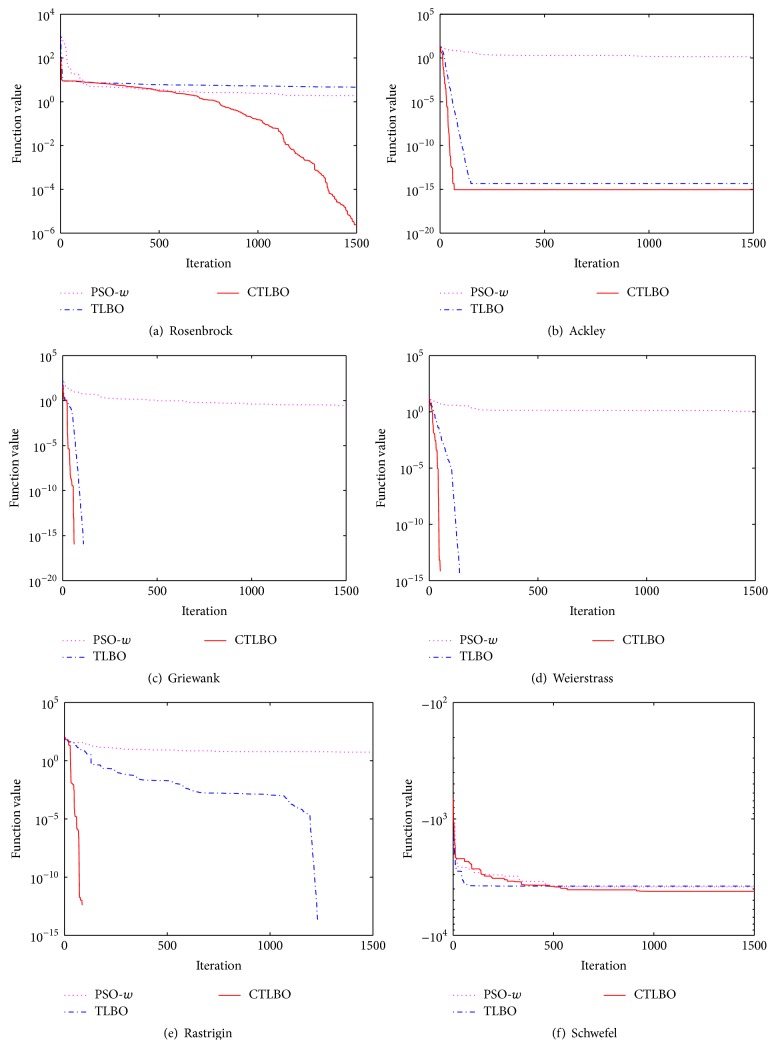
Convergence curve of six functions.

In order to observe the performance of CTLBO visually, the convergence curves of six functions are drawn as shown in Figure 1. To compare CTLBO with other algorithms, we select the convergence curves of PSO-*w* and TLBO to observe their convergence properties.

The Rosenbrock function is always used as a test function to test the performance of optimization algorithms. The global optimum lies inside a long, narrow, parabolic shaped flat valley, and it is very difficult to find the global optimum. It can be seen from Figure 1(a) that the PSO-*w* and TLBO converge with low optimization accuracy, while the CTLBO has good searching ability for the Rosenbrock function.

The Ackley function is a continuous, rotating, and nonseparable multimodal function. The exterior region of the function is nearly flat while the centre is a high peak, and it has many widespread locally optimal points from the flat region to the centre peak. From Figure 1(b) we can see that both TLBO and CTLBO have better performance than the PSO-*w* algorithm. In addition, the convergence rate and accuracy of CTLBO are better than those of TLBO.

It can be observed from Figures 1(c)–1(e) that, for the Griewank, Weierstrass, and Rastrigin functions, TLBO and CTLBO can achieve the global optimum in a very few iterations, while the PSO-*w* can only reach a local optimum. Also, the convergence rate of CTLBO is faster than that of TLBO.

From Figure 1(f), we can see that Schwefel's function is difficult for all three algorithms, which converge with low optimization accuracy.

From the results and analysis, we can see that the proposed TLBO has good searching ability for most functions, and CTLBO has improved its performance. The convergence rate and accuracy of CTLBO are better than those of TLBO. In order to test the proposed algorithm comprehensively, more test functions will be introduced in the next section.

### 4.2. Experiment 2

In this experiment, the performance of the proposed CTLBO algorithm is compared with those of the recently developed PS-ABC [33], TLBO, and I-TLBO. In this part of the work, CTLBO is tested on 13 unconstrained benchmark functions. These functions have no fixed number of dimensions. In other words, the dimension of the problems can be set at will [34]. In this case, we can test the performance of algorithms for high-dimensional problems. The characteristics of these functions are described in Table 3.

This experiment is conducted from small-scale to large-scale by considering 20, 30, and 50 dimensions for all the benchmark functions. The number of function evaluations is set as 120000 for all tested algorithms. Each benchmark function is tested 30 times and the results are obtained in the form of the mean solution and the standard deviation of the objective function after 30 independent runs of the algorithms.

Table 4 shows the comparative results of PS-ABC, TLBO, I-TLBO, and CTLBO algorithms for the 13 functions with 120000 maximum function evaluations.

It can be observed from Table 4 that I-TLBO outperforms the basic TLBO and PS-ABC algorithms for the Quartic, Penalized, and Penalized 2 functions (for all the dimensions) and the Rosenbrock function (for 20 dimensions). PS-ABC outperforms TLBO and I-TLBO for the Rosenbrock (for 30 and 50 dimensions) and Schwefel functions. For the Schwefel 1.2 function, the performances of TLBO and I-TLBO are identical and better than that of the PS-ABC algorithm. The performances of PS-ABC and I-TLBO are identical for the Rastrigin function, while the performances of all three algorithms are identical for the Sphere, Schwefel 2.22, and Griewank functions. For the Ackley function, the performances of PS-ABC and CTLBO are more or less similar.

**Figure 2:**
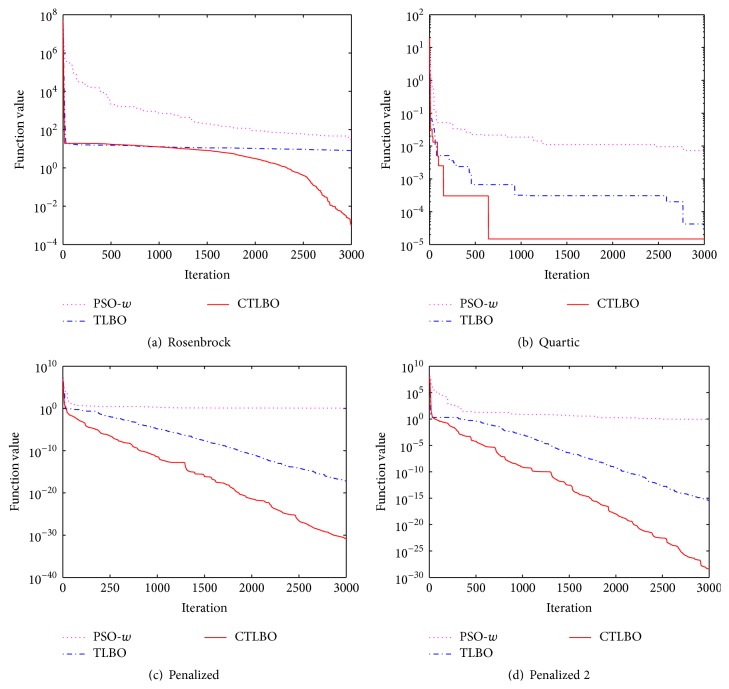
Convergence curve of four functions.

In order to observe the performance of CTLBO visually, the convergence curves of algorithms for several functions in 20 dimensions are drawn as shown in Figure 2.

From Figure 2(a) we can see that, for the Rosenbrock function with 20 dimensions, PSO-*w* and TLBO become trapped in local optima and the search accuracy is very low, while the capability of CTLBO is good and its convergence accuracy is high.

The Quartic function is unimodal with random noise. Noisy functions are widespread in real-world problems, and every evaluation of the function is disturbed by noise, so the algorithms' information is inherited and diffused noisily, which makes the problem hard to optimize. It can be observed from Figure 2(b) that CTLBO has better performance than the other two algorithms.

From Figures 2(c) and 2(d), we can observe that CTLBO has a better searching ability for the Penalized and Penalized 2 functions. CTLBO has better results for these two functions, while TLBO and PSO-*w* are trapped in local optima.

From the above analysis, we can see that the proposed TLBO has good searching ability for most of the functions, and CTLBO has improved its performance based on TLBO. The convergence rate and accuracy of CTLBO show better performance compared to TLBO, which reveals that the proposed chaotic mechanism is effective and provides an improvement on TLBO.

### 4.3. Discussion

This paper formulated a novel TLBO algorithm, based on its combination with chaotic search and Lévy flight. From a quick look, CTLBO resembles other swarm-intelligence approaches such as GA and PSO in many aspects; for example, (1) they are all population-based algorithms and the initial populations are randomly produced; (2) similarly to other evolution strategies, CTLBO has special mutation operators like the teacher phase and the learner phase. However, despite the fact that CTLBO is somewhat similar to other metaheuristics, there are some significant differences between them which help it to outperform other techniques on a number of problems.

It can be seen from the framework of the CTLBO that the population is first divided into two parts in each iteration, then these two subparts evolve with the Lévy flight and teaching-learning mechanisms, respectively, and then the population is perturbed by using chaotic searching. This can be viewed as a kind of coevolution to some extent; that is, two independent subpopulations evolve interactively and, due to this process, not only are the decision solutions diversely exploited but also the convergence rate of the algorithm is accelerated.

Taking a closer look at the CTLBO, we conclude that it essentially consists of three components: exploitation by mutation operator, a global exploration by Lévy flight, and diversification by chaos mapping. The mutation operators including the teacher phase and learner phase ensure the exploitation around the best solution obtained so far. Lévy flight makes the search move away from the worst place with a large step and, at the same time, samples the search space effectively so that the new solutions are thoroughly diversified. Chaos mapping can disturb the solution so as to maintain the population diversity as well as avoid falling into local optima. In general, a good integration of the above three components may thus lead to an efficient algorithm such as CTLBO.

Furthermore, from simulation studies in which the CTLBO algorithm's controlling parameters were varied, we observed that the convergence rate is insensitive to algorithm parameters such as *p*_*a*_. This feature is mainly inherited from the chaotic search. In this process, the randomness with nonzero probability ensures that some of the solutions are discarded and replaced by new ones, which is similar in spirit to the probability of acceptance of worse solutions in the annealing process. From this perspective, it means that there is no need to fine-tune the algorithm parameters of the proposed CTLBO for a specific problem.

Moreover, it can be seen that the proposed method may not find the global minima of a few specific functions. This has its root in the mechanism of the teacher phase in TLBO, where a mean value is used to update the solutions, which may lead solutions directly to the centre of a search region. For those functions whose global minima are not located in the centre of the feasible solution region, it is usually challenging to find the global optima of the tested functions. However, in the CTLBO, the divided population weakens the effect of the “mean mechanism.”

Finally, in this paper merely logistic map has been embedded to diversify the population of CTLBO algorithm; however, other different chaotic maps will be analyzed in the future work.

## 5. Conclusion

This paper proposes chaotic teaching-learning-based optimization with Lévy flight (CTLBO). The algorithm is improved via a Lévy walk and perturbed by chaotic searching, which can enhance the diversification of the algorithm. The experimental results demonstrate that the designed algorithm has better performance than other methods. In addition, the properties of the proposed algorithm are analyzed and the characteristics and features are discussed in the paper.

Future work is likely to apply this novel method to a wider spectrum of problems such as constrained optimization problems and many engineering applications in the real world. What is more, the parallel implementation mechanism of CTLBO and its application to multiobjective optimization as well as combinatorial optimization problems will also be studied.

## Figures and Tables

**Pseudocode 1:**
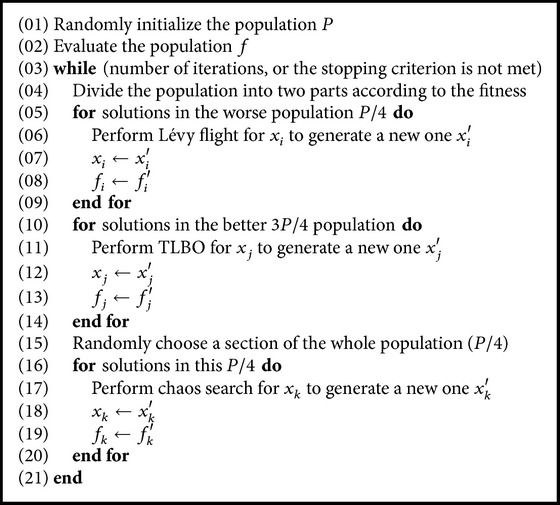
Pseudocode of CTLBO.

**Table 1:** Benchmark functions considered in Experiment 1.

Number	Function	Formulation	Dim.	Search range
1	Sphere	$F_{min}\left(x\right)=\overset{D}{\underset{i=1}{\sum }}x_{i}^{2}$	10	[–100, 100]

2	Rosenbrock	$F_{min}\left(x\right)=\overset{D-1}{\underset{i=1}{\sum }}\left(100{\left(x_{i}^{2}-x_{i+1}\right)}^{2}+{\left(x_{i}-1\right)}^{2}\right)$	10	[–2.048, 2.048]

3	Ackley	$F_{min}\left(x\right)=-20\exp\left(-0.2\sqrt{\frac{1}{30}\overset{D}{\underset{i=1}{\sum }}x_{i}^{2}}\right)-\exp\left(\frac{1}{30}\overset{D}{\underset{i=1}{\sum }}\cos2\pi x_{i}\right)+20+e$	10	[–32.768, 32.768]

4	Griewank	$F_{min}\left(x\right)=\frac{1}{4000}\overset{D}{\underset{i=1}{\sum }}x_{i}^{2}-\prod_{i=1}^{D}\left(\frac{x_{i}}{\sqrt{i}}\right)+1$	10	[–600, 600]

5	Weierstrass	$F_{min}\left(x\right)=\overset{D-1}{\underset{i=1}{\sum }}\left(\overset{k_{max}}{\underset{k=0}{\sum }}\left[a^{k}\cos\left(2\pi b^{k}\left(x_{i}+0.5\right)\right)\right]\right)-D\overset{k_{max}}{\underset{k=0}{\sum }}\left[a^{k}\cos\left(2\pi b^{k}\cdot 0.5\right)\right]\;a=0.5,\;b=3,\;k_{max}=20$.	10	[–0.5, 0.5]

6	Rastrigin	$F_{min}\left(x\right)=\overset{D}{\underset{i=1}{\sum }}\left[x_{i}^{2}-10\cos\left(2\pi x_{i}\right)+10\right]$	10	[–5.12, 5.12]

7	NCRastrigin	$F_{min}\left(x\right)=\overset{D}{\underset{i=1}{\sum }}\left(y_{i}^{2}-10\cos\left(2\pi y_{i}\right)+10\right)\;y_{i}=\left\{\begin{array}{cc} x_{i} & \left|x_{i}\right|<\frac{1}{2}\\ \frac{round\left(2x_{i}\right)}{2} & \left|x_{i}\right|\geq \frac{1}{2} \end{array}\right.\;for\;\;i=1,2,\ldots ,D$.	10	[–5.12, 5.12]

8	Schwefel	$F_{min}\left(x\right)=418.9829\times D-\overset{D}{\underset{i=1}{\sum }}x_{i}\sin\left({\left|x_{i}\right|}^{1/2}\right)$	10	[–500, 500]

**Table 2:** Comparative results of different algorithms over 30 independent runs.

Algorithm	Sphere		Rosenbrock		Ackley		Griewank	
	Mean	SD	Mean	SD	Mean	SD	Mean	SD
PSO-*w*	7.96*E* − 051	3.56*E* − 050	3.08*E* + 000	7.69*E* − 001	1.58*E* − 014	1.60*E* − 014	9.69*E* − 002	5.01*E* − 002
PSO-cf	9.84*E* − 105	4.21*E* − 104	6.98*E* − 001	1.46*E* + 000	9.18*E* − 001	1.01*E* + 000	1.19*E* − 001	7.11*E* − 002
PSO-*w*-local	2.13*E* − 035	6.17*E* − 035	3.92*E* + 000	1.19*E* + 000	6.04*E* − 015	1.67*E* − 015	7.80*E* − 002	3.79*E* − 002
PSO-cf-local	1.37*E* − 079	5.60*E* − 079	8.60*E* − 001	1.56*E* + 000	5.78*E* − 002	2.58*E* − 001	2.80*E* − 002	6.34*E* − 002
UPSO	9.84*E* − 118	3.56*E* − 117	1.40*E* + 000	1.88*E* + 000	1.33*E* + 000	1.48*E* + 000	1.04*E* − 001	7.10*E* − 002
FDR	2.21*E* − 090	9.88*E* − 090	8.67*E* − 001	1.63*E* + 000	3.18*E* − 014	6.40*E* − 014	9.24*E* − 002	5.61*E* − 002
FIPS	3.15*E* − 030	4.56*E* − 030	2.78*E* + 000	2.26*E* − 001	3.75*E* − 015	2.13*E* − 014	1.31*E* − 001	9.32*E* − 002
CPSO-H	4.98*E* − 045	1.00*E* − 044	1.53*E* + 000	1.70*E* + 000	1.49*E* − 014	6.97*E* − 015	4.07*E* − 002	2.80*E* − 002
CLPSO	5.15*E* − 029	2.16*E* − 28	2.46*E* + 000	1.70*E* + 000	4.32*E* − 10	2.55*E* − 014	4.56*E* − 003	4.81*E* − 003
ABC	7.09*E* − 017	4.11*E* − 017	2.08*E* + 000	2.44*E* + 000	4.58*E* − 016	1.76*E* − 016	1.57*E* − 002	9.06*E* − 003
Modified ABC	7.04*E* − 017	4.55*E* − 017	4.42*E* − 001	8.67*E* − 001	3.32**E** − 016	1.84**E** − 016	1.52*E* − 002	1.28*E* − 002
TLBO	**0.00**	**0.00**	1.72*E* + 00	6.62*E* − 01	3.55*E* − 15	8.32*E* − 31	**0.00**	**0.00**
I-TLBO (NT = 4)	**0.00**	**0.00**	2.00*E* − 01	1.42*E* − 01	1.42*E* − 15	1.83*E* − 15	**0.00**	**0.00**
CTLBO	**0.00**	**0.00**	2.47**E** − 02	8.15**E** − 02	8.88*E* − 16	0.00*E* + 00	**0.00**	**0.00**

Algorithm	Weierstrass		Rastrigin		NCRastrigin		Schwefel	
	Mean	SD	Mean	SD	Mean	SD	Mean	SD

PSO-*w*	2.28*E* − 003	7.04*E* − 003	5.82*E* + 000	2.96*E* + 000	4.05*E* + 000	2.58*E* + 000	3.20*E* + 002	1.85*E* + 002
PSO-cf	6.69*E* − 001	7.17*E* − 001	1.25*E* + 001	5.17*E* + 000	1.20*E* + 001	4.99*E* + 000	9.87*E* + 002	2.76*E* + 002
PSO-*w*-local	1.41*E* − 006	6.31*E* − 006	3.88*E* + 000	2.30*E* + 000	4.77*E* + 000	2.84*E* + 000	3.26*E* + 002	1.32*E* + 002
PSO-cf-local	7.85*E* − 002	5.16*E* − 002	9.05*E* + 000	3.48*E* + 000	5.95*E* + 000	2.60*E* + 000	8.78*E* + 002	2.93*E* + 002
UPSO	1.14*E* + 000	1.17*E* + 00	1.17*E* + 001	6.11*E* + 000	5.85*E* + 000	3.15*E* + 000	1.08*E* + 003	2.68*E* + 002
FDR	3.01*E* − 003	7.20*E* − 003	7.51*E* + 000	3.05*E* + 000	3.35*E* + 000	2.01*E* + 000	8.51*E* + 002	2.76*E* + 002
FIPS	2.02*E* − 003	6.40*E* − 003	2.12*E* + 000	1.33*E* + 000	4.35*E* + 000	2.80*E* + 000	7.10*E* + 001	1.50*E* + 002
CPSO-H	1.07*E* − 015	1.67*E* − 015	0	0	2.00*E* − 001	4.10*E* − 001	2.13*E* + 002	1.41*E* + 002
CLPSO	**0**	**0**	**0**	**0**	**0**	**0**	**0**	**0**
ABC	9.01*E* − 006	4.61*E* − 005	1.61*E* − 016	5.20*E* − 016	6.64*E* − 017	3.96*E* − 017	7.91*E* + 000	2.95*E* + 001
Modified ABC	0.00**E** + 000	0.00**E** + 000	1.14*E* − 007	6.16*E* − 007	1.58*E* − 011	7.62*E* − 011	3.96*E* + 000	2.13*E* + 001
TLBO	2.42*E* − 05	1.38*E* − 20	6.77*E* − 08	3.68*E* − 07	2.65*E* − 08	1.23*E* − 07	2.94*E* + 02	2.68*E* + 02
I-TLBO (NT = 4)	**0.00**	**0.00**	**0.00**	**0.00**	**0.00**	**0.00**	1.10*E* + 02	1.06*E* + 02
CTLBO	**0.00**	**0.00**	**0.00**	**0.00**	**0.00**	**0.00**	1.99*E* + 02	1.26*E* + 02

*Source*. The results of algorithms other than TLBO, I-TLBO, and CTLBO are taken from [32]. The results of TLBO and I-TLBO are taken from [15].

**Table 3:** Benchmark functions considered in Experiment 2.

Number	Function	Formulation	Search range
1	Sphere	$F_{min}\left(x\right)=\overset{D}{\underset{i=1}{\sum }}x_{i}^{2}$	[−100, 100]

2	Schwefel 2.22	$F_{min}\left(x\right)=\overset{D}{\underset{i=1}{\sum }}\left|x_{i}\right|+\prod_{i=1}^{D}\left|x_{i}\right|$	[−10, 10]

3	Schwefel 1.2	$F_{min}\left(x\right)=\overset{D}{\underset{i=1}{\sum }}{\left(\overset{i}{\underset{j=1}{\sum }}x_{j}^{2}\right)}^{2}$	[−100, 100]

4	Schwefel 2.21	*F*_min_(*x*) = max⁡(|*x*_*i*_|)	[−100, 100]

5	Rosenbrock	$F_{min}\left(x\right)=\overset{D-1}{\underset{i=1}{\sum }}\left(100{\left(x_{i}^{2}-x_{i+1}\right)}^{2}+{\left(x_{i}-1\right)}^{2}\right)$	[−30, 30]

6	Step	$F_{min}=\overset{D}{\underset{i=1}{\sum }}{\left(\left\lfloor x_{i}+0.5\right\rfloor \right)}^{2}$	[−100, 100]

7	Quartic	$F_{min}\left(x\right)=\overset{D}{\underset{i=1}{\sum }}ix_{i}^{4}+\mathrm{rand}\left[0,1\right)$	[−1.28, 1.28]

8	Schwefel	$F_{min}\left(x\right)=418.9829\times D-\overset{D}{\underset{i=1}{\sum }}x_{i}\sin\left({\left|x_{i}\right|}^{1/2}\right)$	[−500, 500]

9	Rastrigin	$F_{min}\left(x\right)=\overset{D}{\underset{i=1}{\sum }}\left[x_{i}^{2}-10\cos\left(2\pi x_{i}\right)+10\right]$	[−5.12, 5.12]

10	Ackley	$F_{min}\left(x\right)=-20\exp\left(-0.2\sqrt{\frac{1}{30}\overset{D}{\underset{i=1}{\sum }}x_{i}^{2}}\right)-\exp\left(\frac{1}{30}\overset{D}{\underset{i=1}{\sum }}\cos2\pi x_{i}\right)+20+e$	[−32.768, 32.768]

11	Griewank	$F_{min}\left(x\right)=\frac{1}{4000}\overset{D}{\underset{i=1}{\sum }}x_{i}^{2}-\prod_{i=1}^{D}\left(\frac{x_{i}}{\sqrt{i}}\right)+1$	[−600, 600]

12	Penalized	$F_{min}=\frac{\pi }{D}\left[10\;\sin^{2}\left(\pi y_{1}\right)+\overset{D-1}{\underset{i=1}{\sum }}{\left(y_{i}-1\right)}^{2}\left[1+10\;\sin^{2}\left(\pi y_{i+1}\right)\right]+{\left(y_{D}-1\right)}^{2}\right]+\overset{D}{\underset{i=1}{\sum }}u\left(x_{i},10,100,4\right)$ $y_{i}=1+\frac{x_{i}+1}{4};\;u\left(x_{i},a,k,m\right)=\left\{\begin{array}{cc} k{\left(x_{i}-a\right)}^{m} & x_{i}>a\\ 0 & -a<x_{i}<a\\ k{\left(-x_{i}-a\right)}^{m} & x_{i}<-a \end{array}\right.$	[−50, 50]

13	Penalized 2	$\begin{array}{c} F_{min}=0.1\left\{\sin^{2}\left(3\pi x_{1}\right)+\overset{D-1}{\underset{i=1}{\sum }}{\left(x_{i}-1\right)}^{2}\left[1+\sin^{2}\left(3\pi x_{i+1}\right)\right]+{\left(x_{D}-1\right)}^{2}\left[1+\sin^{2}\left(2\pi x_{D}\right)\right]\right\} \end{array}+\overset{D}{\underset{i=1}{\sum }}u\left(x_{i},5,100,4\right)$ $u\left(x_{i},a,k,m\right)=\left\{\begin{array}{cc} k{\left(x_{i}-a\right)}^{m} & x_{i}>a\\ 0 & -a<x_{i}<a\\ k{\left(-x_{i}-a\right)}^{m} & x_{i}<-a \end{array}\right.$	[−50, 50]

**Table 4:** Comparative results of different algorithms over 30 independent runs.

Function	Dim.	PS-ABC [33]		TLBO [15]		I-TLBO [15]		CTLBO	
		Mean	SD	Mean	SD	Mean	SD	Mean	SD
Sphere	20	**0.00**	**0.00**	**0.00**	**0.00**	**0.00**	**0.00**	**0.00**	**0.00**
	30	**0.00**	**0.00**	**0.00**	**0.00**	**0.00**	**0.00**	**0.00**	**0.00**
	50	**0.00**	**0.00**	**0.00**	**0.00**	**0.00**	**0.00**	**0.00**	**0.00**

Schwefel 2.22	20	**0.00**	**0.00**	**0.00**	**0.00**	**0.00**	**0.00**	**0.00**	**0.00**
	30	**0.00**	**0.00**	**0.00**	**0.00**	**0.00**	**0.00**	**0.00**	**0.00**
	50	**0.00**	**0.00**	**0.00**	**0.00**	**0.00**	**0.00**	**0.00**	**0.00**

Schwefel 1.2	20	1.04*E* + 03	6.11*E* + 02	**0.00**	**0.00**	**0.00**	**0.00**	**0.00**	**0.00**
	30	6.11*E* + 03	1.69*E* + 03	**0.00**	**0.00**	**0.00**	**0.00**	**0.00**	**0.00**
	50	3.01*E* + 04	4.11*E* + 03	**0.00**	**0.00**	**0.00**	**0.00**	**0.00**	**0.00**

Schwefel 2.21	20	0.00	0.00	**0.00**	**0.00**	**0.00**	**0.00**	**0.00**	**0.00**
	30	8.59*E* − 115	4.71*E* − 114	4.9*E* − 324	**0.00**	**0.00**	**0.00**	**0.00**	**0.00**
	50	19.6683	6.31*E* + 00	9.9*E* − 324	**0.00**	**0.00**	**0.00**	**0.00**	**0.00**

Rosenbrock	20	0.5190	1.08*E* + 00	15.0536	2.28*E* − 01	1.3785	8.49*E* − 01	3.04**E** − 03	3.03**E** − 03
	30	**1.5922**	4.41**E** + 00	25.4036	3.50*E* − 01	15.032	1.2*E* + 00	11.1767	9.40*E* − 01
	50	**34.4913**	3.03**E** + 01	45.8955	2.89*E* − 01	38.7294	7.57*E* − 01	36.9081	1.15*E* + 00

Step	20	2.61*E* − 16	3.86*E* − 17	9.24*E* − 33	4.36*E* − 33	**0.00**	**0.00**	**0.00**	**0.00**
	30	5.71*E* − 16	8.25*E* − 17	1.94*E* − 29	1.88*E* − 29	**0.00**	**0.00**	**0.00**	**0.00**
	50	1.16*E* − 15	1.41*E* − 16	3.26*E* − 13	5.11*E* − 13	1.51*E* − 32	8.89*E* − 33	**0.00**	**0.00**

Quartic	20	6.52*E* − 03	2.25*E* − 03	1.07*E* − 02	5.16*E* − 03	5.16*E* − 03	4.64*E* − 03	1.86**E** − 04	2.72**E** − 04
	30	2.15*E* − 02	6.88*E* − 03	1.15*E* − 02	3.71*E* − 03	5.36*E* − 03	3.72*E* − 03	1.62**E** − 04	1.15**E** − 04
	50	6.53*E* − 02	1.77*E* − 02	1.17*E* − 02	5.00*E* − 03	5.60*E* − 03	3.40*E* − 03	1.48**E** − 04	1.44**E** − 04

Schwefel	20	**−8379.66 **	4.72**E** − 12	−8210.23	1.66*E* + 02	−8263.84	1.16*E* + 02	−7817.05	2.46*E* + 02
	30	**−12564.23 **	2.55**E** + 01	−12428.60	1.53*E* + 02	−12519.92	1.16*E* + 02	−10903.20	3.95*E* + 02
	50	**−20887.98 **	8.04**E** + 01	−20620.72	1.89*E* + 02	−20700.70	1.64*E* + 02	−15744.41	6.51*E* + 02

Rastrigin	20	**0.00**	**0.00**	6.41*E* − 14	6.16*E* − 14	**0.00**	**0.00**	**0.00**	**0.00**
	30	**0.00**	**0.00**	6.95*E* − 13	1.64*E* − 12	**0.00**	**0.00**	**0.00**	**0.00**
	50	**0.00**	**0.00**	7.90*E* − 13	1.89*E* − 12	**0.00**	**0.00**	**0.00**	**0.00**

Ackley	20	8.88*E* − 16	0.00	3.55*E* − 15	8.32*E* − 31	7.11**E** − 16	**0.00**	8.88*E* − 16	0.00
	30	8.88*E* − 16	0.00	3.55*E* − 15	8.32*E* − 31	7.11**E** − 16	**0.00**	8.88*E* − 16	0.00
	50	8.88*E* − 16	0.00	3.55*E* − 15	8.32*E* − 31	7.11**E** − 16	**0.00**	8.88*E* − 16	0.00

Griewank	20	**0.00**	**0.00**	**0.00**	**0.00**	**0.00**	**0.00**	**0.00**	**0.00**
	30	**0.00**	**0.00**	**0.00**	**0.00**	**0.00**	**0.00**	**0.00**	**0.00**
	50	**0.00**	**0.00**	**0.00**	**0.00**	**0.00**	**0.00**	**0.00**	**0.00**

Penalized	20	2.55*E* − 16	4.97*E* − 17	4.00*E* − 08	6.85*E* − 24	2.42*E* − 16	1.09*E* − 16	3.41**E** − 32	1.99**E** − 32
	30	5.53*E* − 16	8.68*E* − 17	2.67*E* − 08	6.79*E* − 12	4.98*E* − 16	2.14*E* − 16	6.08**E** − 22	2.48**E** − 21
	50	1.02*E* − 15	1.58*E* − 16	5.18*E* − 05	1.92*E* − 04	9.19*E* − 16	5.38*E* − 16	3.13**E** − 16	5.41**E** − 16

Penalized 2	20	2.34*E* − 18	2.20*E* − 18	2.34*E* − 08	6.85*E* − 24	1.93*E* − 18	1.12*E* − 18	2.26**E** − 19	8.29**E** − 19
	30	6.06*E* − 18	5.60*E* − 18	2.37*E* − 08	4.91*E* − 10	5.92*E* − 18	4.74*E* − 18	3.80**E** − 18	1.51**E** − 18
	50	5.05*E* − 17	1.53*E* − 16	1.52*E* − 03	5.29*E* − 03	4.87*E* − 17	4.26*E* − 17	2.13**E** − 17	2.09**E** − 17
